# *HCC1*, a Polygalacturonase, Regulates Chlorophyll Degradation via the Ethylene Synthesis Pathway

**DOI:** 10.1186/s12284-023-00675-8

**Published:** 2023-12-09

**Authors:** Yongxiang Liao, Bing Xiang, Zhenzhen Xue, Asif Ali, Yong Li, Mengyuan Li, Aiji Wei, Jialu Xin, Daiming Guo, Yingxiu Liao, Yunfeng Tian, Zhixue Zhao, Peizhou Xu, Hongyu Zhang, Xiaoqiong Chen, Yutong Liu, Hao Zhou, Duo Xia, Kangxi Du, Xianjun Wu

**Affiliations:** State Key Laboratory of Crop Gene Exploration and Utilization in Southwest China, Rice Research Institute, Sichuan Agricultural University, Chengdu, 611130 China

**Keywords:** Chlorophyll degradation, D-galacturonic acid, Ethylene, High chlorophyll content, Polygalacturonase

## Abstract

Chlorophyll degradation is an important physiological process and is essential for plant growth and development. However, how chlorophyll degradation is controlled at the cellular and molecular level remains largely elusive. Pectin is a main component of the primary cell wall, and polygalacturonases (PGs) is a group of pectin-hydrolases that cleaves the pectin backbone and release oligogalacturonide. Whether and how PGs affect chlorophyll degradation metabolism and its association with ethylene (ETH) have not been reported before. Here, we report a novel function of PG in a mutant ‘*high chlorophyll content1’ hcc1*, which displayed a decrease in growth and yield. Our morphological, biochemical and genetic analyses of *hcc1*, knockout lines and complementation lines confirm the function of *HCC1* in chlorophyll degradation. In *hcc1*, the PG activity, ETH content and D-galacturonic acid (D-GA) was significantly decreased and showed an increase in the thickness of the cell wall. Exogenous application of ETH and D-GA can increase ETH content and induce the expression of *HCC1*, which further can successfully induce the chlorophyll degradation in *hcc1*. Together, our data demonstrated a novel function of *HCC1* in chlorophyll degradation via the ETH pathway.

**Supplementary Information:**

The online version contains supplementary material available at 10.1186/s12284-023-00675-8.

## Introduction

Pectin is a component of the cell wall and its synthesis, modification and degradation metabolism affect the fluidity and extensibility of plant growth and development (Anderson 2016). Synthesis and degradation of pectin should be in balance for the proper functioning of the cell wall. Pectin is a complex molecule composed of sugars and its most abundant domain is homogalacturonan (HG). In addition to HG, Rhamnogalacturonan I (RGI) and (RGII) are also found in the cell wall (Harholt et al. 2010; Mohnen 2008). HG is a long-chain macromolecule composed of α-D-galacturonic acid monomers, which are linked with different sugars and methyl and acetyl groups as side chains. HG is synthesized in the Golgi apparatus, then secreted to cell wall in methyl-esterified and acetylated forms, which are later degraded or modified by cell wall enzymes (Kim and Brandizzi 2014; Toyooka et al. 2009). The degradation and modification of pectin are accomplished by a series of pectin hydrolyzing enzymes. Polygalacturonase (PGs) are pectin hydrolyzing enzymes that cleave the glycosidic bonds of galacturonic acid and are involved in the breakdown of the pectin network (Biely et al. 1996). PGs have been reported to play their role in fruit ripening, organ morphogenesis and organ abscission. Methyl-esterification and demethyl-esterification of pectin are important for the degradation specificity of pectin hydrolyzing enzymes. Pectin methylesterases (PMEs) remove the methyl-esters group from HG and change the synthesized methyl-esterified pectin to demethyl-esterified pectin. While pectin methyl-esterases inhibitors (PMEIs) reverse the activity of PMEs. Blockwise demethyl-esterified HG molecule can increase the stiffness of cell wall by crosslinking with calcium, however random demthyl-esterified can be further cleaved with pectate lyases (PL) and PGs.

Pectin modification enzymes affect plant growth and development by regulating cell wall remodeling and cell proliferation and stiffness. Cell proliferation, expansion and differentiation are the important cytological basis in plant growth and development (Albersheim et al. 1996). In *Arabidopsis, ARABIDOPSIS DEHISCENCE ZONE POLYGALACTURONASE1* (*ADPG1)* and *ADPG2* encode PGs that are required for cell division and expansion in reproductive development (Ogawa et al. 2009). *POLYGALACTURONASE INVOLVED IN EXPANSION1* (*PGX1*) is involved in cell proliferation and expansion and regulates floral development in *Arabidopsis* (Xiao et al. 2014). *POLYGALACTURONASE45 (PG45)* hydrolyzes pectin and regulates leaf curvature by cell wall proliferation (Yang et al. 2021). In *Malus domestica* (apple), the overexpression of *MdPG1* causes abnormal leaf morphology and fruit shedding, however downregulating the expression of *MdPG1* enhances cell adhesion and reduces cell proliferation in fruit (Poles et al. 2020). In rice, the *PHOTO-SENSITIVE LEAF ROLLING 1 (PSL1)* encoding a *PG* modifies cell wall biosynthesis and participates in drought response and leaf morphogenesis (Zhang et al. 2021). In addition, *OsBURP16* encodes a β-subunit of PG1 and its overexpression plants displayed enhanced activity, decreased pectin, and increased sensitivity to abiotic stress (Liu et al. 2014).

Chlorophyll (chl) is a photosynthetic pigment, present in the thylakoid membrane, which absorbs light energy and converts it into chemical energy. Usually, the synthesis and degradation of chl are in a state of dynamic equilibrium in the plants. Changes in chl degradation rate are the indicator of plant growth and development, for example, senescence, cell death, biotic and abiotic stresses could promote its degradation (Benedetti et al. 1998; Gräfe et al. 1999). The degradation of chl during senescence is essential as its products are reused as a source of nitrogen and could repress oxidation to maintain cell viability (Ginsburg and Matile 1993; Hörtensteiner 2006). Therefore, chl degradation has an important biological function in plants. It starts with the conversion of chl *b* to chl *a,* which normally occurs with help of different chlorophyllase, and is influenced by temperature, light, and environmental stimuli. Ethylene (ETH) is an important phytohormone and is involved in ripening and senescence-related traits by modulating the expression of different transcription factors (Fu et al. 2021). In *Citrus sinensis ETHYLENE RESPONSE FACTOR13 (ERF13)* has been reported to be involved in the degreening of fruit peel and chl degradation (Yin et al. 2016). *PHYTOCHROME INTERACTING 4 (PIF4)*, *PIF5*, *MdPUB24, ETHYLENE INSENSITIVE 3 (EIN3)* and *PHEOPHORBIDE A OXYGENASE (PAO)* have also been reported to regulate the senescence by chl catabolism (Qiu et al. 2015; Song et al. 2014; Wei et al. 2021; Zhang et al. 2015).

The leaf-color mutants (LCMs) are ideal genetic material for exploring chlorophyll metabolism, photosynthesis, degradation and senescence. Several LCMs have been isolated and characterized in *Arabidopsis* (Ren et al. 2007; Sakuraba et al. 2012), maize (Greene et al. 1988; Li et al. 2021) and rice (Chen et al. 2020; Miyoshi et al. 2003). In rice, more than 120 LCMs-related genes have been cloned, and a few of those mutants were called stay green, delayed yellowing, and high chlorophyll content etc. A deeper understanding of these leaf color related genes attracts the attention of molecular breeders as their potential for increasing yield. These genes encode a subunit of the CCAAT-box-binding transcription factor (Feng et al. 2014), a chlorophyll *b* reductase (Kusaba et al. 2007; Morita et al. 2009), an α/β hydrolase-fold family protein (Morita et al. 2009), a magnesium ion chelatase (Jiang et al. 2007; Müller et al. 2007), a golden2-like transcription factor (Nakamura et al. 2009) and so on. Therefore, the molecular mechanism of leaf coloration in plants is regulated by a complicated regulatory network. Although a variety of LCM-related genes have been characterized, however, whether pectin metabolism is affected in chlorophyll degradation and how PG modulates chlorophyll levels in rice remains to be defined.

In the current study, we identified a rice mutant “*higher chlorophyll content 1* (*hcc1*)” that displayed higher chlorophyll content. Genetic analysis showed that the *HCC1* encodes a member of the PG family and regulates the composition and structure of the cell wall. The genetic complementation and loss-of-function of *HCC1* demonstrated that *HCC1* affects pectin metabolism of the plant cell wall, which leads to high chlorophyll phenotype and reduced plant growth. The blade leaves of *hcc1* showed decreased contents of D-GA and ETH and exogenous supplementation of D-GA can increase ETH content and promote the expression of *HCC1*. Together, our data indicated that *HCC1* encodes a PG that regulates pectin metabolism and chlorophyll degradation via ETH synthesis pathway.

## Materials and Methods

### Plant Materials and Growth Conditions

The mutant *hcc1* was generated using ethyl methane sulfonate (EMS) treatment of a Chinese *indica* cultivar Yixiang1B (*Oryza sativa* L.), one of the elite backbone parents in hybrid rice breeding programs in China, and is the corresponding maintainer line of Yixiang1A. Yixiang1B was taken as wild type (WT) throughout the study, and both WT and *hcc1* were grown alternatively in the paddy fields in Sichuan, Chengdu (N30.67°, E104.06°) and Hainan, Lingshui (N18.47°, E110.04°), in China.

### Measurement of Chlorophyll Contents and Photosynthetic Parameters

Fresh leaves (0.1 g) were placed in a mortar containing 2 mL of distilled water and ground into a homogenate. And then dilute to 10 mL with distilled water. Take 1.5 mL of sample solution, add 6 mL acetone solution (80%), let it stand for stratified. The resulting upper solution was the chlorophyll acetone solution, which was examined by a spectrophotometer at 663 and 645 nm. Then, the content of chlorophylls was calculated as follows:

$${\text{Chlorophyll}}\;\left( {{\text{Chl}}} \right)\;{\text{a}}\;{\text{concentration}}\;\left( {{\text{mg}}/{\text{L}}} \right) = 12.7 \times {\text{OD}}_{663} - \, 2.69 \times {\text{OD}}_{645}$$

$${\text{Chlorophyll}}\;\left( {\text{Chl }} \right)\;{\text{b}}\;{\text{concentration}}\;\left( {{\text{mg}}/{\text{L}}} \right) = 22.9 \times {\text{OD}}_{645} - 4.68 \times {\text{OD}}_{663}$$

Photosynthesis parameters (net photosynthetic rate, transpiration rate, and stomatal conductance, etc.) were measured using a portable photosynthetic apparatus (Li-6400, Li-Cor, NE, United States). All measurements were carried out using the parameters mentioned in the previous study by Zhang et al. (2021).

### RNA Extraction and qRT-PCR

Total RNA was extracted using Trizol (Invitrogen, Carlsbad, CAs, United States) following the instruction of the manufacturer. The mRNA was digested with DNase I (Invitrogen, Carlsbad, CA, United States) and was subjected to reverse transcription to synthesize first-stand cDNA. Oligo (dT) primer and SuperScript II (Invitrogen, Carlsbad, CA, United States) was used for the reverse transcription. The qRT-PCR analysis was performed using a Bio-Rad CFX96 Real-Time System coupled to a C1000 Thermal Cycler (Bio-Rad, Hercules, CA, United States). *OsActin* was used as the internal control. The sequences of primers used in the relative expression were listed in Additional file 1: Table S1, S2.

### Genetic Analysis and Map-Based Cloning

Two populations were derived by crossing wild type × *hcc1* and *hcc1* × wild type and were used for genetic analysis. Two F_2_ populations were also derived from the reciprocal cross of a *japonica* cv. 02428 and *hcc1* and were mapping the candidate gene controlling the phenotype of *hcc1*. For the bulk segregation analysis (BSA) leaf blades from 10 F_2_ individual plants were collected for DNA extraction to construct the *hcc1* and WT DNA pools, respectively. The physical linkage map was constructed using molecular markers for screening polymorphism in *hcc1*. The SSR primers were synthesized according to the Gramene database (http://www.gramene.org/microdat). InDel markers were developed based on the alignment results of the reference genome of 93–11 (an *indica* cv.) and the Nipponbare (http://rise2.genomics.org.cn/page/rice/index.jsp), (a *japonica* cv.) genome sequence around the primary region. Primers were designed using Primer3 web version 4.0.0. The specificity of each primer was confirmed by BLAST and PCR analysis. The sequences of SSR and InDel markers were listed in Table S3. For Mutmap analysis, *hcc1* was backcrossed with the WT and then self-crossed to generate BC_1_F_2_ population. The DNA of 20 BC_1_F_2_ individual plants were pooled and sequenced using a facility provided by Novogene Corporation (Beijing, China). Mutmap was performed, according to the instruction of Abe et al., to find the candidate genes showing the highest SNP index (Table S4). PCR amplified products were separated on 3.0% agarose gel in 0.5 × TBE buffer and visualized and photographed under UV light.

### Vector Construction and Transformation

A CRISPR/cas9 vector was constructed to knock out *HCC1*. Briefly two targets, hcc1*-*Y (CTCAAGGTGCAGAACAGCC) and hcc1*-*B (TCTCCTACTCCGGCATCCGCCG) with protospacer adjacent motif (PAM) sequence “CCG” were identified from the coding sequence of *HCC1*. Oligos were designed and possible off-targets were prevented using BLAST search and knock-out constructs were amplified using PCR according to a previous study (Ali et al. 2022). Endonuclease, *Eco*311 and T4 ligase, the hcc1-Y was inserted into the pBWA(V)-cas9i2 and the hcc1-B were inserted into the pBWD(LB)DNAi. Finally, pBWA(V)-cas9i2-hcc1-Y and pBWD(LB)DNAi-hcc1-B were assembled into the final vector pBWA(V)-cas9i2-hcc1 using endonuclease Sapi and T4 ligase. For the complementation, a vector containing a 2 kb native promoter and complete gDNA sequence of *HCC1* along with 500 bp downstream was constructed and assembled into pCAMBIA1300. The primers were designed by CE Design using a pair of primers (F: 5′-CGGGGTACCCGATGCGCCTCGATGATTCC-3′, R: 5′-CCGGAATTCGAGGCAATGAGGCCATCTAG-3′). The PCR amplifications endonucleases *Eco*RI and *Bam*HI using were performed according to the previous study of Peng et al. (2018). The constructs were verified by sequencing and the recombinant mixture containing recombinant constructs was transformed into *E.coli* following the guidelines of *Agrobacterium*-mediated transformation of Ma et al. (2016). Positive clones were transformed into the calli using the guidelines of Toki et al. (2006). The sequence of positive knock-out lines was verified from genomic DNA from T_1_ transgenic plants using a primer (F: 5′-CCTAACGCAACGACCTTTT-3′, R: 5′-CTGGAACGTCTACGGCAAC-3′).

### Determination of PG Activity

PG activity was measured from leaves at particular leaf stages developmental stages according to the previous study by Zhang et al. (Zhang et al. 2021). PG activity was tested using a PG detection kit (BC2660) purchased from Solarbio Science and Technology Co., Ltd (Beijing, China), using D-GA as internal standard. Briefly, 0.2 g fresh leaves were soaked in 1 mL of extract solution and the sample was ground into homogenate and centrifuged at 16,000 g for 10 min at 4℃. 50 μL supernatants were taken and reacted with D-GA for 2 h. The 200 μL reaction solution was used to measure the absorbance at 540 nm. The PG activity was calculated as follows:

$$PG\;activity\;(U/g) = X*V/W/T = \frac{0.5X}{W}$$

X: value derived from a standard curve (μmol/mL); V: volume of extract added, 1 mL; W: sample weight (g); T: time of enzymatic reaction, 2 h.

### Morphological Observation of Cell Wall

Leaves were soaked in 2.5% glutaraldehyde for 12 h and then moved into 1.0% osmium anhydride stationary liquid, followed by dehydration with the concentration gradient (30%, 50%, 70%, 80%, 90 and 100%) of propyl-alcohol. After epoxy penetration, ultrathin sections were double stained with uranium acetate and lead citrate, and samples were observed under a transmission electron microscope (HITACHI-HT7700, Tokyo, Japan).

### Ethylene Assay

Ethylene (ETH) content were measured using the enzyme-linked immunosorbent assay (ELISA) previously described by Teng et al. (2006). ETH content were extracted using the ETH detection kit (BC-286) following the procedures of the manufacturer, which was purchased from Solarbio Science and Technology Co., Ltd (Beijing, China). Briefly, draw the standard curve according to the instructions. 1 g fresh leaves were soaked in 4 mL of extract solution and the sample was ground into homogenate and centrifuged at 10,000 g for 20 min at 4℃. Add the standard diluent 50 μL and sample extract solution 50 μL and incubate at 37℃ for 30 min. Then, add conjugate reagent 50 μL and incubate, wash. After adding the chromogenic solution and incubate at 37℃ for 10 min, the reaction was terminated using stop solution for 15 min and absorbance was measured at 450 nm. A standard curve was drawn with the standard concentration as abscissa and OD value as the ordinate. Construct the linear regression equation and obtain the sample concentration. The results are subsequently multiplied by the dilution factor, which was the actual concentration of the sample.

### Dark-Induced Leaf Senescence

Leaves were cut into 1 cm pieces, and 20–30 pieces were placed in a 50 mL petri dish. The various treatment fluids (30 mL, ethephon 300 mg/L, D-GA 100 mg/L, and H_2_O as control) were respectively injected into the Petri dishes. The duration of dark treatment was 4 d at 28℃.

### Determination of D-Galacturonides (D-GA) Content

D-GA content were measured using high-performance liquid chromatography (HPLC) as follows. The leaves from *hcc1* (with obvious mutant phenotypes) were selected at the mature stage and the same growth stage was selected from that of WT. 20 mg leaves were soaked in 1 mL polysaccharide solution and 1 mL 4 mol/L TFA for 5 h in a 110℃ oven, followed by 3–5 times distillation, obtained 2 mg of dry matter and dissolved in 200 μL of 0.3 mol/L NaOH solution, and then added 200 μL 0.5 mol/L PMP for 60 min in 70℃. After the reaction, 200 μL 0.3 mol/L HCl and 1 mL chloroform were added, and then the sample was centrifuged at 250 g for 5 min. The supernatant was filtered through a 0.22 μm membrane and the absorbance was measured at 540 nm. Then, the content of D-GA was calculated as follows:

$$\omega = \frac{\rho \times V \times 1000}{{m \times 1000}} \times n$$

$\rho$: the concentration of D-GA in the sample solution (mg/L); *V:* the final volume of sample (mL); m: the sample weight (g); n: the dilution factor.

## Results

### *hcc1* Showed Increased chl Contents and Reduced Expression of chl-Degradation Related Genes

**Fig. 1:**
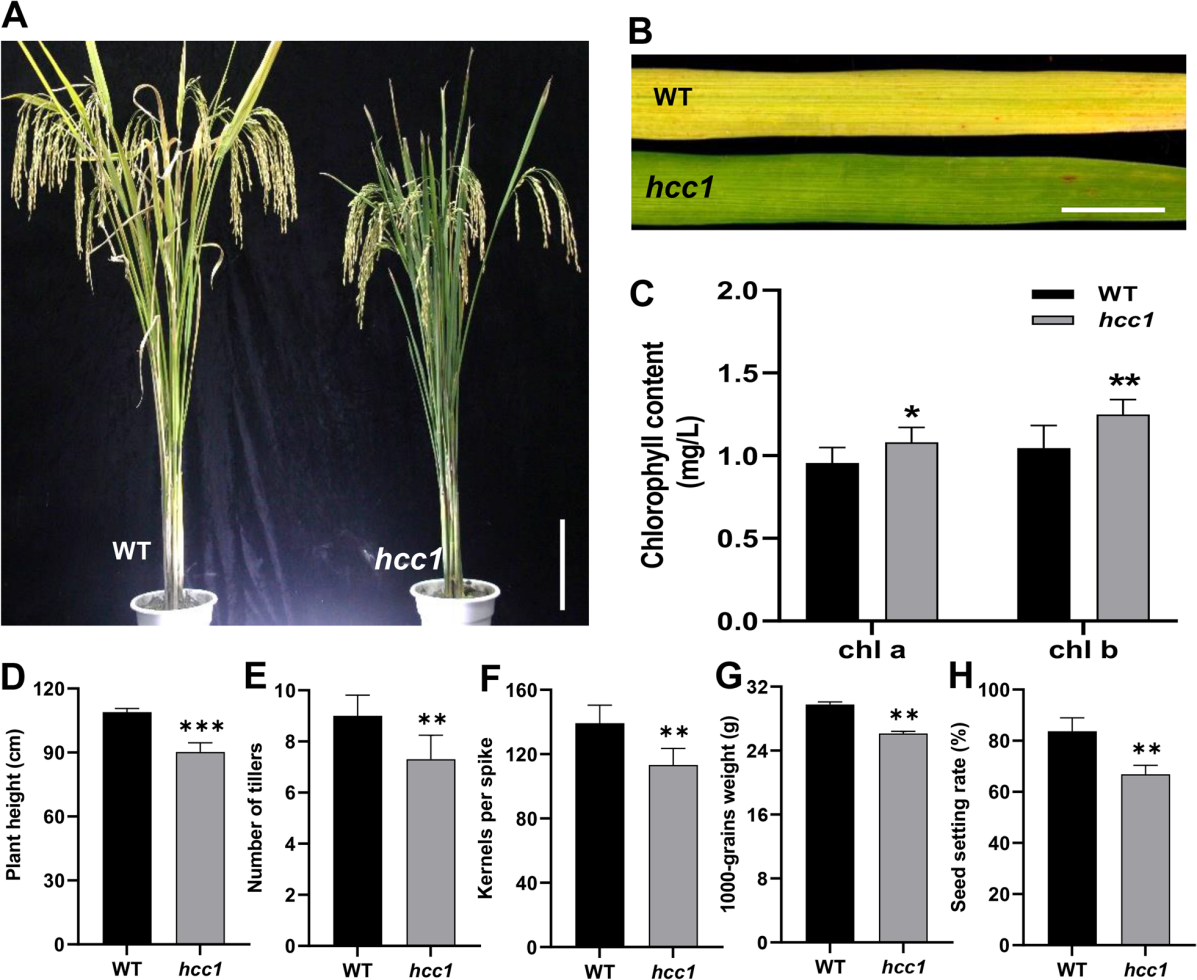
Phenotypic observation of wild type and *hcc1*. **A** WT and *hcc1* at maturity. **B** The phenotype of WT and *hcc1* leaves at maturity. Scale bar in **A** = 20 cm and in **B** = 2 cm. **C** Comparison of chlorophyll a and chlorophyll b contents between WT and *hcc1* at mature stage. Mean and SD were obtained from three independent experiments. Comparison of plant height (**D**), number of tillers (**E**), kernels per spike (**F**), 1000-grains weight (**G**), and seed setting rate (**H**) between WT and *hcc1* respectively. Data presented in **D**–**G** were the average of n = 10 plants. Statistical analysis was performed using two-tailed Student’s *t*-test, *, ** and ***indicate *p* < 0.05, *p* < 0.01 and *p* < 0.001 respectively

To explore the genetic mechanism of chlorophyll metabolism, a mutant (*hcc1)* was screened from the leaf-color mutants group from an EMS-mutagenized population of Yixiang1B (*indica* cv.), which was used as wild type (WT) throughout the study. WT showed the yellowing of leaves at maturity, which is normal, however, *hcc1* showed a stable deep-green leaf phenotype (Fig. 1A, B). To characterize the deep-green leaf phenotype of *hcc1*, we carried determined the chl contents. The result indicated the significantly increased chlorophyll a and chlorophyll b contents of *hcc1* compared to WT at the mature stage, and SPAD value of hcc1 is higher than that of WT (Fig. 1C, Additional file 1: Fig. S1). A comparison of agronomic traits with WT revealed that *hcc1* showed a significant reduction in plant height, number of tillers, kernels per spike, 1000-grains weight, and seed setting rate (Fig. 1D–H). *hcc1* also showed a phenotype of withered leaf tip, similar to that of previously reported *psl1*, suggesting *hcc1* is an allelic mutant to *psl1*. However, in current study, we only focused on the novel phenotype of chl contents. Phenotypic analysis revealed that *hcc1* displayed increased contents of chl and retarded growth compared to WT.

**Fig. 2:**
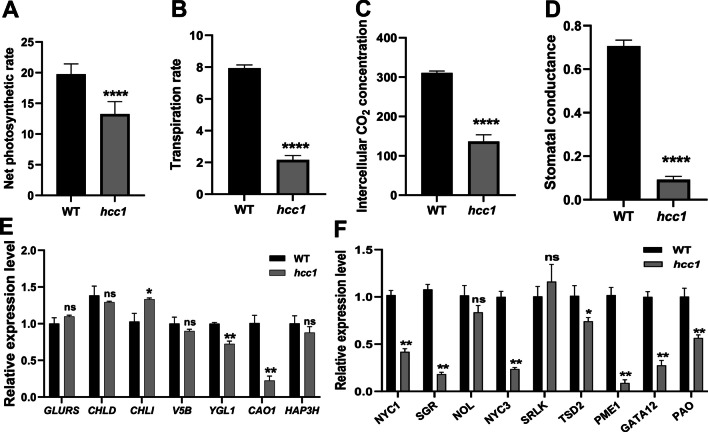
Photosynthetic parameters and the expression of chlorophyll synthesis and degradation-related genes in WT and *hcc1*. **A**–**D** Comparison of net photosynthetic rate (**A**), transpiration rate (**B**), intercellular CO_2_ concentration (**C**) and stomatal conductance (**D**) between WT and *hcc1* at heading stage. **E** The relative expression level of chlorophyll synthesis-related genes at the heading stage. **F** The relative expression level of chlorophyll degradation-related genes at the heading stage. Mean and SD were obtained from three independent measurements. Statistical analysis was performed using two-tailed Student’s *t*-test, *, ** and **** indicate *p* < 0.05, *p* < 0.01 and *p* < 0.0001, respectively and “ns” represents no significant difference

To explore the relationship of increased chlorophyll content with photosynthesis in *hcc1*, we tested the photosynthesis parameters in leaf blades at the heading stage. Net photosynthetic rate, transpiration rate, intercellular CO_2_ concentration and stomatal conductance of *hcc1* were significantly decreased compared to WT (Fig. 2A–D). To dissect further the reduction in growth and photosynthetic parameters associated with chl synthesis or degradation, the relative expression of chlorophyll synthesis and degradation-related genes were analyzed. The relative expression level of chlorophyll synthesis-related genes in *hcc1* was not significantly different compared to WT (Fig. 2E). However, the expression of chl degradation-related genes was significantly downregulated in *hcc1* (Fig. 2F). These results revealed that the chlorophyll degradation was repressed in *hcc1*, which resulted in chlorophyll enrichment but did not enhance the photosynthesis parameters.

### Genetic Analysis of *hcc1*

To find the candidate gene responsible for *hcc1* phenotype, two populations were developed by crossing *hcc1* with *japonica* cv. 02428 and WT (*indica* cv.) in reciprocal crosses. The former mapping population was used for SSR and gene mapping, while later was used to study the genetic behavior of the candidate gene. All F_1_ plants did not reveal a deep-green leaf phenotype on leaf blades and statistics shown in Table 1 revealed that 1/3^rd^ of the F_2_ population showed the deep-green leaf phenotype. Chi-square analysis of segregation rations between WT and *hcc1* fits with the Mendelian ratio of 3:1. Hence, the deep-green leaf phenotype of *hcc1* was controlled by a single recessive nuclear gene.

**Table 1:** Segregation ratios of F_2_ plants with and without LCM phenotype

Population	Total Plants Observed	Plants with WT’ Phenotype	Plants with mutant’ Phenotype	Segregation ratio	χ2
*hcc1/*WT	702	521	181	2.87	0.69
WT*/hcc1*	665	496	169	2.93	1.12

**Fig. 3:**
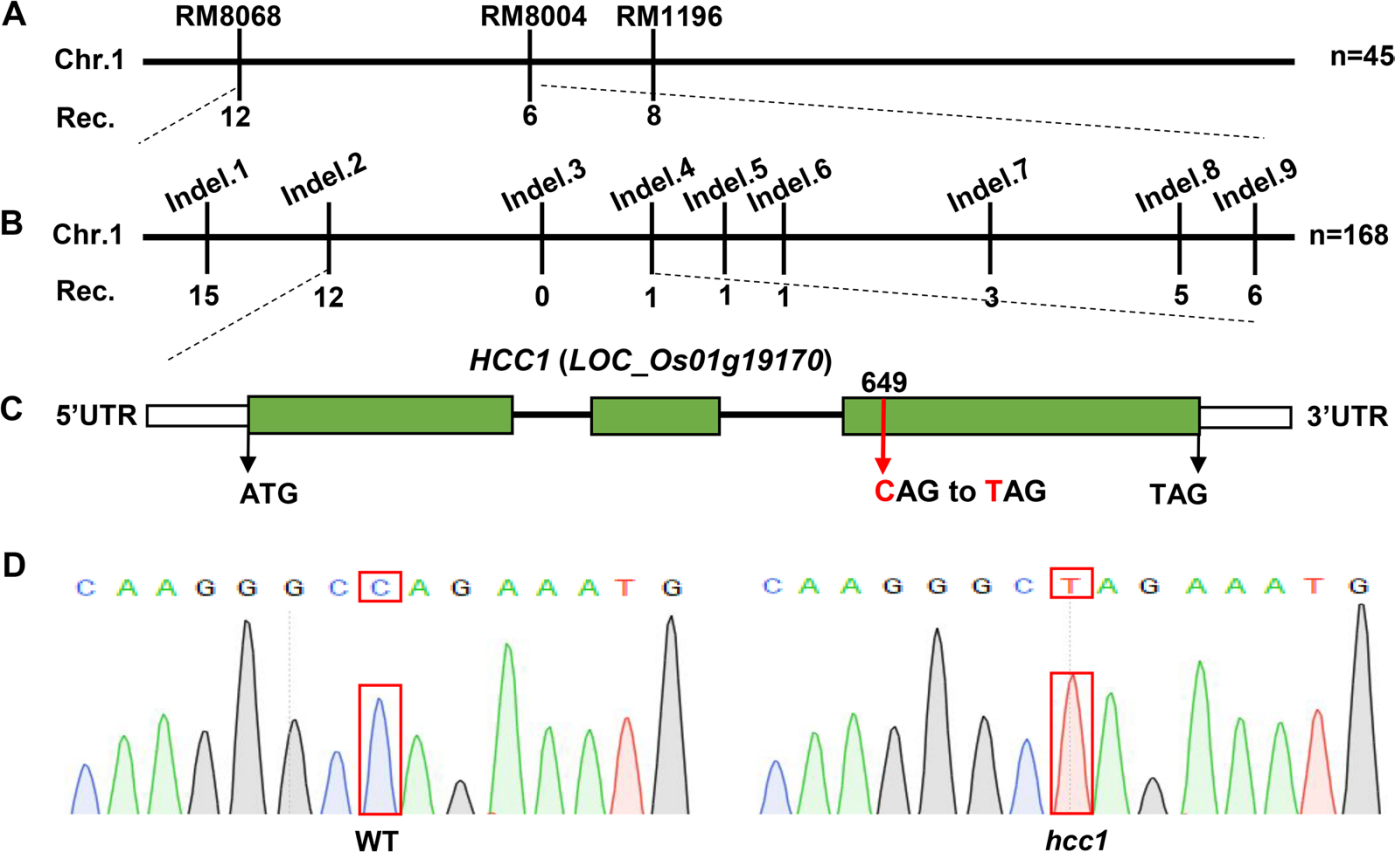
SSR marker analysis and gene mapping of candidate gene of *hcc1*. **A** The position of the candidate gene of *hcc1* on chromosome 1 between SSR marker RM8068 and RM8004. **B** A further narrowed down region of the candidate gene of *hcc1* between Indel marker Indel.2 and Indel. 4. **C** The structure of the candidate gene, exons and introns are indicated by green rectangles and black lines respectively. **D** Chromatograms showing the sequence comparison of WT and *hcc1*

To find the candidate gene, the mapping population (*hcc1* × 02428) was subjected to SSR analysis. DNA of 45 F_2_ plants showing *hcc1* phenotype was pooled and screened for polymorphism using 700 SSR markers distributed uniformly on all chromosomes of the rice genome. Initial screening found that molecular markers (RM8068, RM8004 and RM1196) on chromosome 1 were co-segregated with the mutant phenotype (Fig. 3A). Linkage analysis indicated that the candidate gene was mapped to a 600 kb interval between the InDel marker Indel.2 and Indel.4, co-segregated with Indel.3 (Fig. 3B and Table S3). Due to the further unavailability of markers, MutMap analysis was performed by using the bulked DNA sample from 25 individuals of BC_1_F_2_ population with a deep-green leaf phenotype according to a previous study by Abe et al. (Abe et al. 2012). MutMap revealed a single base mutation with an SNP index of 1 localized on the 649^th^ base in the third exon of *LOC_Os01g19170* (Additional file 1: Fig. S2 and Table S4)*.* Individual comparison of sequence chromatograms confirmed the presence of an SNP (C > T) in *hcc1*. A single base substitution (C > T) caused a change in 110th amino acid by altering the genetic codon from CAG to TAG, which is a stop codon (Fig. 3C, D and Table S5). These results indicated that *hcc1* phenotype was likely to be due to the premature termination of protein of LOC_Os01g19170*.* According to the MSU rice database, this gene encodes a polygalacturonase (PG) and hence we tentatively named it *HCC1*.

**Fig. 4:**
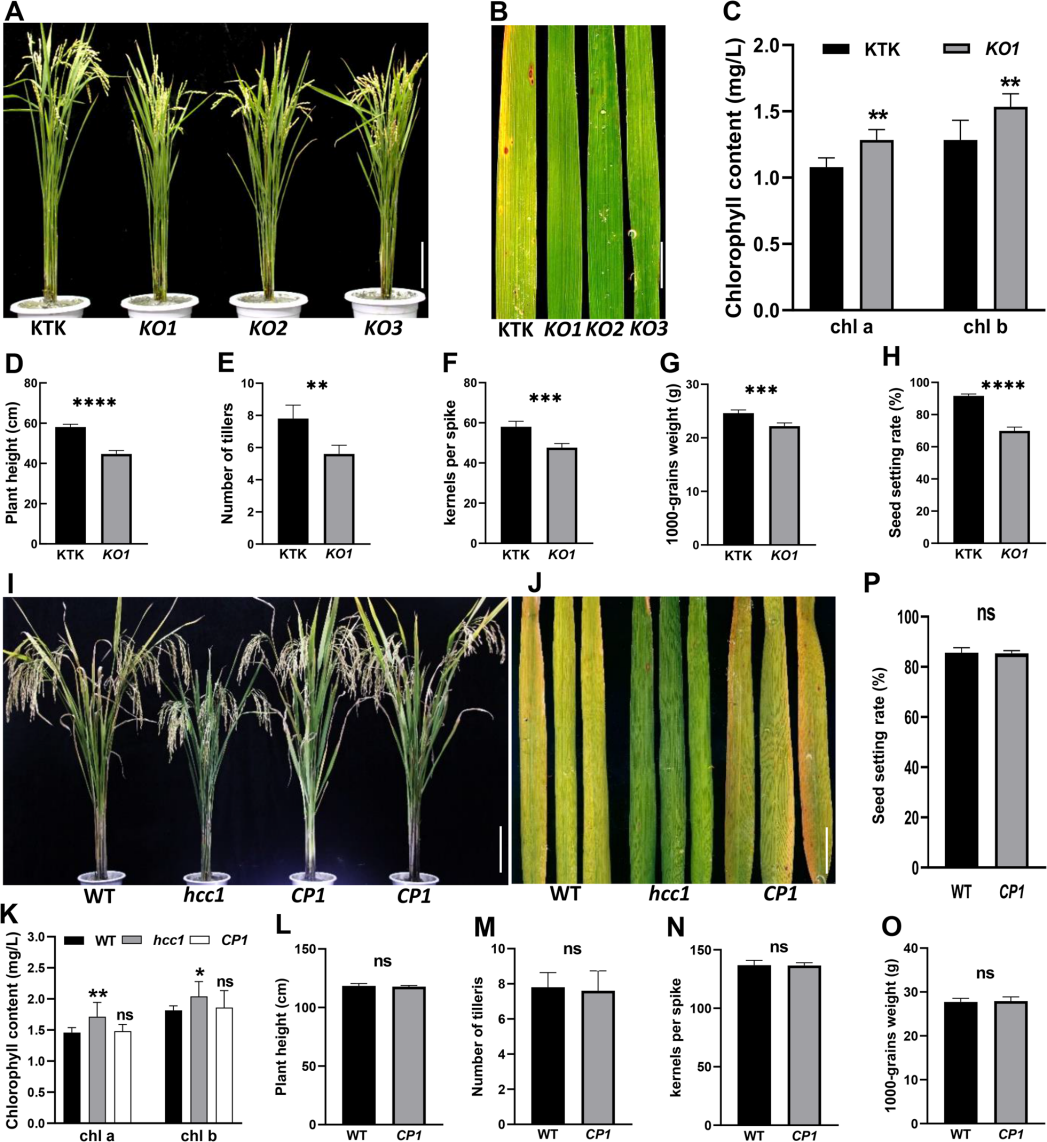
Functional validation of candidate gene. **A** The morphology of knockout (*KO*) lines and Kitaake (KTK) at the mature stage. **B** The deep-green leaf phenotype of *KO* lines at maturity. **C** Comparison of leaves chlorophyll a and chlorophyll b contents of WT and *KO1* at the maturity stage. Mean and SD were obtained from three independent experiments. Comparison of plant height (**D**), number of tillers (**E**), kernels per spike (**F**), 1000-grains weight (**G**) and seed setting rate (**H**) between KTK and *KO1* respectively. **I** The morphology of complementary lines (*CP*) at maturity. **J** The leaf blade color of WT, *hcc1* and *CP1* at maturity. **K** Comparison of leaves chlorophyll a and chlorophyll b contents at maturity stage between WT, *hcc1* and *CP1.* Mean and SD were obtained from three independent experiments. **L**–**P** showed plant height, number of tillers, kernels per spike, 1000-grains weight and seed setting rate between WT and *CP1* respectively. Mean and SD were obtained from three independent measurements in **C** and **K**. Data presented in **D**–**H**, **L**–**P** are the average of n = 10 plants. Statistical analysis was performed using two-tailed Student’s *t*-test, *, **, ***, ****indicate *p* < 0.05, *p* < 0.01, *p* < 0.001 and *p* < 0.0001, “ns” indicates no significant difference. Scale bar in **A**, **I** = 20 cm, and in **B**, **J** = 2 cm

To confirm phenotype in *hcc1* was controlled by *HCC1*, a CRISPR/Cas9 vector, targeting the exonic region of *HCC1,* was introduced into the calli of Kitaake (KTK, *japonica* cv.). Three independent knockout lines (KO) were developed, which showed the deep-green leaf phenotype similar to that of *hcc1* (Fig. 4A, B, Additional file 1: Fig. S3A–E). *KO1* also displayed significantly higher contents of chl and SPAD value compared to KTK (Fig. 4C, Additional file 1: Fig. S4) and reduced agronomic traits comparable to *hcc1* (Fig. 4D–H). To further confirm, *hcc1* was complemented with gDNA of *HCC1.* Complementation lines (*CP*) lines have a fully rescued phenotype of *hcc1* and did not reveal deep-green leaf phenotype (Fig. 4I, J, Additional file 1: Fig. S3F–H). In addition, the chl contents of (Fig. 4K), and agronomic traits (Fig. 4L–P) of *CP1* were comparable to that of WT. Taken together, these results confirmed that the loss-of-function of *HCC1* resulted in the high chlorophyll content phenotype of *hcc1*.

### Phylogenetic Analysis of HCC1

To understand the evolutionary origin and domestication history of HCC1, these species, *Volvox carteri*, *Physcomitrium patens, Selaginella moellendorffii, Amborella trichopoda and* angiosperm, were selected to construct an evolutionary tree and analyze conserved domain. The results indicated that the conserved domain was not identified in *Volvox carteri*, but multiple sequence alignment indicated the partial sequences of algae were similar to the conserved domains. The domains appear to be conserved in *Physcomitrium patens*, and as evolution progresses, the number of conserved domains decreased from 5 to 3 before angiosperms appear. In angiosperms, HCC1 protein has 5–7 conserved domains and both the number and composition of domains become stable (Additional file 1: Fig. S5). These data showed HCC1 may already exist in algae, but its sequence was significantly different. As species evolved, the structure of HCC1 tended to be stable. In angiosperms, HCC1’s structure becomes more conservative and stable.

The sequence alignment analysis of HCC1 protein sequence showed low similarity with different species e.g. similarity indexes between rice and wheat, *Zea mays* were 25.1% and 26.7% respectively (Additional file 1: Fig. S6). The results of the domain architecture analysis revealed that the HCC1 protein has five conserved domains of parallel beta-helix (PBH), which indicates it encodes for a PG. However, the mutation of *hcc1* was not located within the conserved domains.

### HCC1 Alters the Cell Wall Composition and PG Activity

**Fig. 5:**
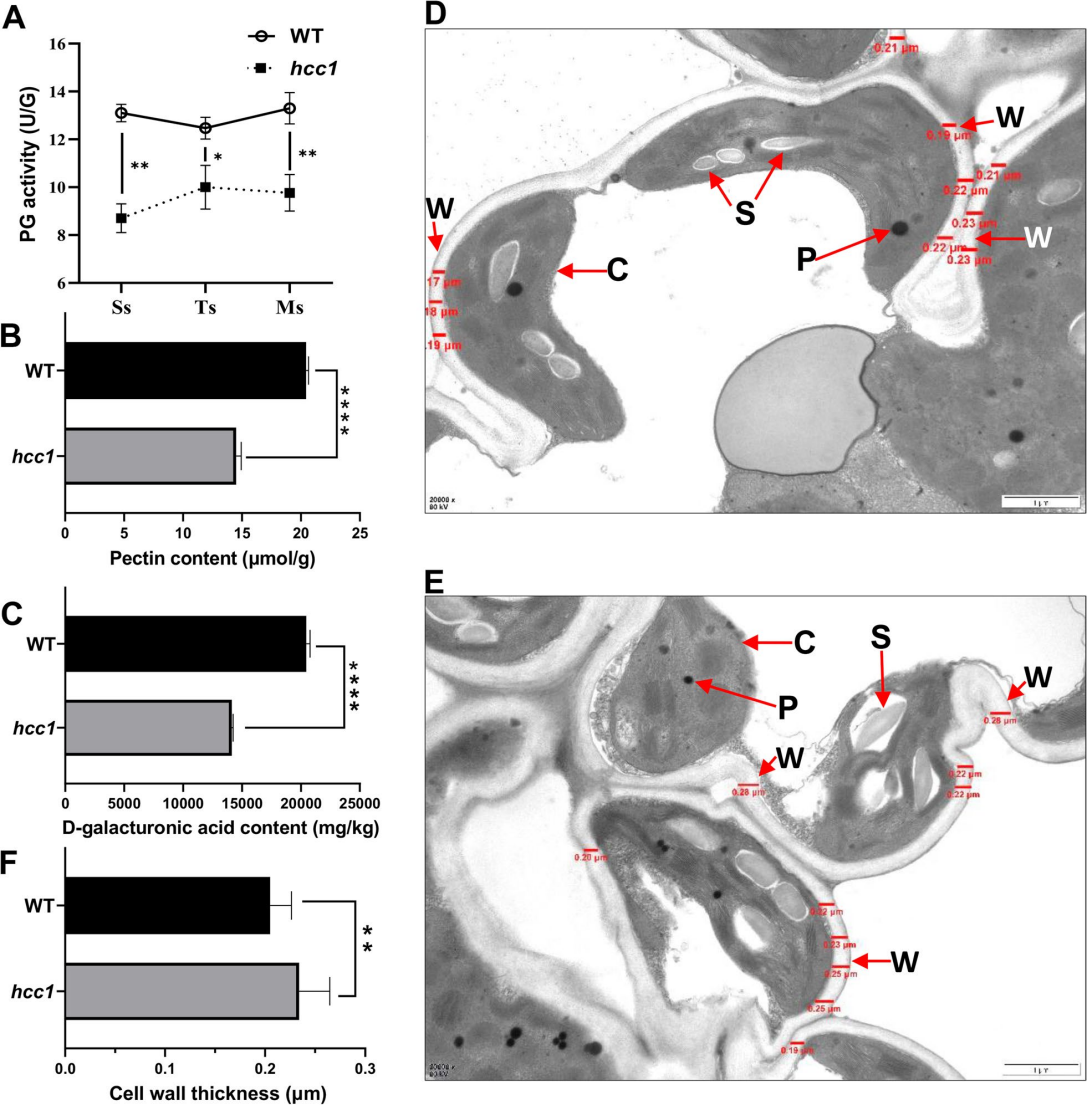
PG activity and structural analysis of cell wall in *hcc1*. **A** Changes of PG activity in the leaf of WT and *hcc1* at the seedling stage (Ss), tillering stage (Ts) and mature stage (Ms). **B** Comparison of total pectin content in the leaf blade between WT and *hcc1* at Ms. **C** Comparison of D-GA content in the leaves between WT and *hcc1* at Ms. **D** TEM showing the subcellular structure of leaf of WT and **E** *hcc1* at Ms. Where C, chloroplast; P, plastoglobule; S, starch grain; and W, cell wall. Scale bar in (D, E) = 1 μm. **F** Comparison of cell wall thickness of WT and *hcc1* in the leaf at Ms. Mean and SD were obtained from three independent measurements (**A**–**C**). The values in (**F**) are the mean ± SD of 10 different determinations (red word in images of **D** and **E**). Statistical analysis was performed using two-tailed Student’s *t*-test, *, ** and **** indicate *p* < 0.05, *p* < 0.01 *p* < 0.0001 and respectively

PG promotes the degradation of the cell wall by cleaving the backbone of pectin (Cooley and Yoder 1998). To determine whether it has affected pectin metabolism, the activity of PG in leaves of *hcc1* and WT was compared at the seedling stage (Ss), tillering stage (Ts) and maturity stage (Ms). The result indicated that PG activity in *hcc1* was significantly lower than WT at all the detected stages (Fig. 5A). The pectin content in leaf blades of *hcc1* was also significantly decreased at the mature stage (Fig. 5B). D-galacturonic acid (D-GA) is the product of PG hydrolyzes the pectin. Quantification and high-performance liquid chromatography (HPLC) analyses revealed that the D-GA contents in *hcc1* were significantly lower compared to its WT (Fig. 5C, Additional file 1: Fig. S7). Observation of mesophyll cells micrographs by transmission electron microscopy (TEM) revealed changes in the thickness of the cell wall. Measurement of the size revealed a significant increase in the width of the cell wall of *hcc1* compared to WT (Fig. 5D–F). Taken together, these results of mutation of *HCC1* affected the cell wall composition and decreased the PG activity.

### Metabolism of Ethylene (ETH) was Changed in* hcc1*

**Fig. 6:**
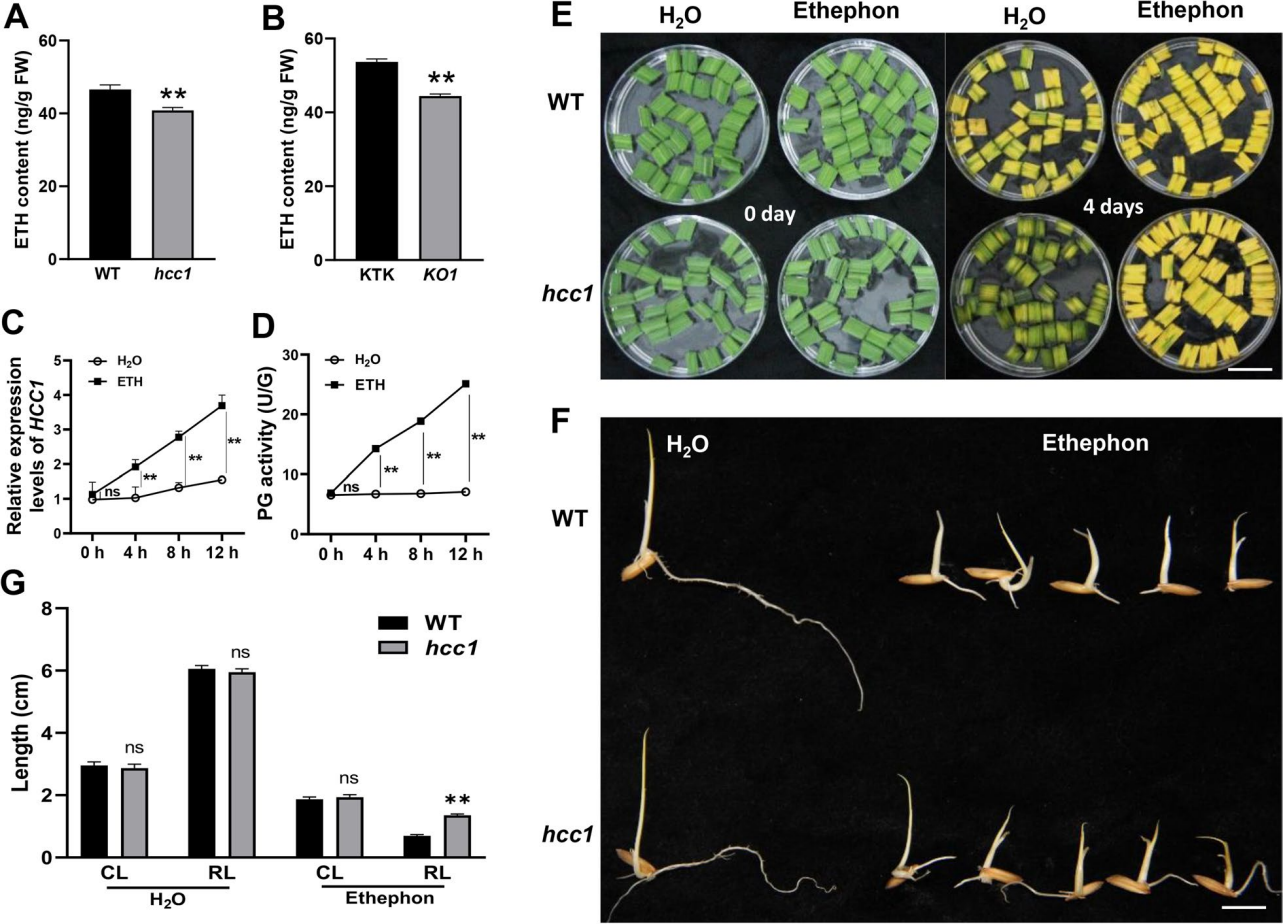
Metabolism and response of WT and *hcc1* to ethylene. **A** Comparison of ETH content in the leaves of WT and *hcc1* at the seedling stage (Ss). **B** Comparison of ethylene contents in the leaves of KTK and *KO1* at the seedling stage. **C** Changes in the relative expression of *HCC1* after ETH treatment in WT at the Ss. Water was treated as contronl. **D** Comparison of PG activity in the WT leaves after ETH treatment at the Ss. Water was treated as contronl. **E** Leaf blades senescence phenotype of WT and *hcc1* after ethephon treatment (300 mg/L) under dark conditions at the Ss. Water was treated as contronl, 4 days at room temperature 28 ℃, Scale bar = 5 cm. **F** Ethylene response phenotypes of seeds of WT and *hcc1*. Water was treated as control, 4 days after seed germination at room temperature 28 ℃. Scale bar = 1 cm. **G** Coleoptile length (CL) and root length (RL) of WT and *hcc1* in response to ethephon (300 mg/L). Data presented in G is the average of 20 seedlings. Mean and SD were obtained from three independent measurements in image **A**–**D**. Statistical analysis was performed using two-tailed Student’s *t*-test, ** indicates* p* < 0.01, and “ns” indicates no significant difference

Previous studies reported that the expression of *PG* genes are induced by ETH in plants (Sitrit and Bennett 1998; Wang et al. 2000, 2020). To test whether the expression of *HCC1* is affected by ETH or not? The ETH contents were measured in the leaf blades in *hcc1, KO1* and WT at the seedling stage. The result indicated that the ETH contents of *hcc1* and *KO1* were significantly lower than that of their WT (Fig. 6A, B). In addition, the expression of major ETH synthesis-related genes, *1-aminocyclopropeane-1-carboxylic acid synthetase* (*ACSs*) and *1-aminocyclopropane-1-carboxylic acid oxidase* (*ACOs*) were also significantly down-regulated in *hcc1* (Additional file 1: Fig. S8). To explore further the relationship between the *PG* expression and ETH, we applied ethephon (a slow ETH-releasing agent) onto the seedlings of WT plants. The expression of *HCC1* and PG activity was gradually upregulated with the time after treatment (Fig. 6C, D). In contrast, after 1-methylcyclopropene (1-MCP) treatment that is an inhibitor of ethylene synthesis, the ETH content, PG activity and expression of *HCC1* were gradually downregulated as time goes by (Additional file 1: Fig. S9). To further confirm, the response of ETH, ethephon was applied to *hcc1* and WT and observed at 0 and 4 days after treatment and water was used as a control. After 0 days, the treatment with water and ethephon retained a deep-green leaf phenotype in *hcc1* and WT. After 4 days, the senescence was induced in the leaves of WT treated with both H_2_O and ethephon. However, the leaf blades of *hcc1* showed senescence phenotype with the treatment of ethephon but not with H_2_O (Fig. 6E). It showed *hcc1* was an ETH-deficient-mutant and the deep-green leaf phenotype could be gotten rid of with exogenous application of ETH. Ethephon was applied to *hcc1* and WT seeds in the dark for the germination process, where H_2_O was used as a control. After 3 days of treatment, the coleoptile length and root length of *hcc1* with treated H_2_O were not significantly different compared to WT. However, the root length of *hcc1* treated ethephon was significantly longer than WT (Fig. 6F, G). These results revealed that the mutation of *HCC1* changed ETH metabolism.

### D-Galacturonic Acid (D-GA) Promotes ETH Synthesis and Induces the Expression of *HCC1*

**Fig. 7:**
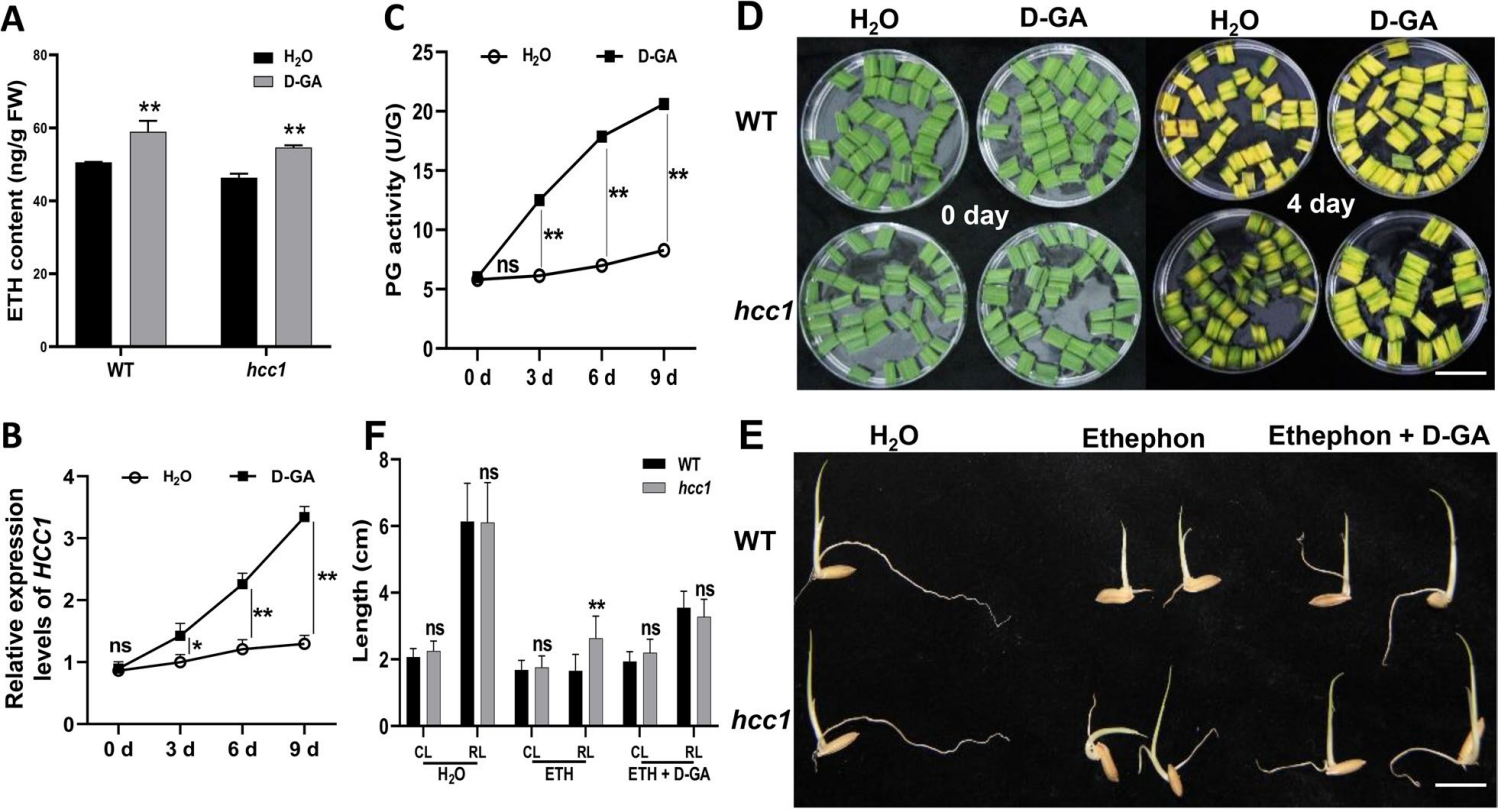
Changes of ethylene response of WT and *hcc1* with D-galacturonic acid treatment. **A** Changes of ethylene contents after D-galacturonic acid (D-GA) treatment after 9 days in WT and *hcc1* at the seedling stage (Ss). **B** Changes of the relative expression levels of *HCC1* after D-GA application in WT at the Ss. Water was treated as control. **C** Changes of PG activity in the leaves after D-GA application at the Ss in WT Water was treated as control. **D** Leaf blades senescence phenotype of WT and *hcc1* after D-GA (100 mg/L) treatment under dark-induced conditions at the Ss. Water was treated as contronl. 4 days at room temperature 28 ℃. **E** Ethylene response phenotypes of seed germination of WT and *hcc1*. Water was treated as control. 4 days after seed germination at room temperature 28 ℃. **F** Coleoptile length (CL) and root length (RL) of WT and *hcc1* in response to ethephon (300 mg/L). Mean and SD were obtained from three independent measurements. Statistical analysis was performed using two-tailed Student’s *t*-test, * and ** indicate *p* < 0.05, *p* < 0.01, respectively, and “ns” indicates no significant difference. Scale bar in **D** = 5 cm, in **E** = 1 cm

D-GA is a monomer and is produced when PG hydrolyzes the pectin. Previous results revealed that D-GA content in leaf blades of *hcc1* were significantly lower compared to WT (Fig. 5C). Previous studies reported that D-GA acts as a signaling molecule and takes part in the regulation of ETH metabolism (Yang et al. 2021). We applied D-GA onto the WT and *hcc1* at the seedling stage and indicated that ETH contents in leaves increased and *HCC1* expression and activity of PG also increases as time passes (Fig. 7A–C). Results revealed, similar to the application of ethephon, after the 4 days of D-GA application onto the leaves of *hcc1* removed the deep-green leaf phenotype (Fig. 7D). However, the leaves showed a consistent deep-green leaf phenotype with the application of H_2_O at both 0 and 4 days of treatment. We treated the ethephon + D-GA onto the *hcc1* and WT seeds in dark during the germination, while ethephon was used as a control (Fig. 7E). After treatment for 3 days, the root length of *hcc1* treated ethephon was significantly longer than that of WT. However, the treatment of ethephon + D-GA did not show a significant increase in the root length of *hcc1* (Fig. 7F). These data suggested that D-GA promoted ETH synthesis and affected leaf blade senescence.

## Discussion

Polygalacturonases are important pectin hydrolases, which have been reported to affect plant growth, fruit ripening, and biotic and abiotic stress response by regulating cell wall metabolism (Atkinson et al. 2002; Liu et al. 2014; Ogawa et al. 2009)). A recent study reported that the loss of the PG, in *psl1*, leads to leaf rolling and enhances drought tolerance (Zhang et al. 2021). Leaf rolling phenotype was regulated in *psl1* due to abnormalities in the bulliform cells of leaves. Here, we identified a *hcc1*, an allelic mutant of *psl1*, which exhibited retarded growth and showed a lack of chl degradation in its leaves at maturity (Fig. 1). Mutant *hcc1* showed more severe losses in terms of yield due to more chl contents. This indicated that the decrease in chl at maturity is essential for maintaining yield.

PGs participate in cell wall remodeling and are involved in the hydrolysis of pectin (Vorwerk et al. 2004). In *Arabidopsis*, *POLYGALACTURONASE45* cleaves pectin affects cell proliferation and regulates leaf blade morphology (Yang et al. 2021) and altered expression of *PGX1* leads to extra petals (Xiao et al. 2014). In this study, the *hcc1* displayed decreased PG activity, reduced pectin content and its hydrolysate D-GA content accompanied with decreased expression of chl degradation-related genes (Figs. 2 and 5). PGs function as pectin hydrolase that cleaves HG and produces oligogalacturonides (OG) and D-GA. The pectin content and PG activity in leaves of *hcc1* were found significantly decreased (Fig. 5A, B), and these results were consistent with the *psl1* mutant, which also showed defects the cell wall structure of bulliform cells (Zhang et al. 2021). Observation of a significant increase in the cell wall thickness in leaves speculated that loss of PG resulted in the loosening of the cell wall (Fig. 5D–F). However, the role of *PSL1* on PG activity and cell wall composition was already reported. Hence we mainly focused on the association of the deep-green leaf phenotype with the ethylene pathway only.

Chlorophyll synthesis and degradation are in a dynamic equilibrium in the plants. The chlorophyll contents of *hcc1* were higher than that of WT at maturity and the expression of chlorophyll degradation pathway genes was significantly repressed in *hcc1* (Figs. 1C and 2E, F). These findings were similar to previously characterized leaf color mutants (Jiang et al. 2007; Kusaba et al. 2007; Morita et al. 2009). Increasing the chl usually employs the increase in the conversion of light energy to chemical energy. Therefore high chlorophyll content may promote grain yield, for example, a deep-green leaf variety in maize enhances the ability to capture light and increase grain yield (Tollenaar et al. 2004). However, a significant increase in *hcc1* revealed that higher chl contents at maturity decrease the photosynthetic efficiency (Figs. 1C and 2A–D). These findings were similar to *ncy1* and *sgr* (Jiang et al. 2007; Kusaba et al. 2007), hence it also demonstrates that high chl contents do not necessarily increase grain yield.

Leaf senescence is usually accompanied by chlorophyll degradation (Quirino et al. 2000). ETH is considered an important hormone in senescence (Yin et al. 2016) *ETHYLENE INSENSITIVE 3* (*EIN3*), an important transcription factor of ETH signaling pathway in *Arabidopsis*, regulates chl degradation by interacting with *PAO*, *NYE1* and *NYC1* (Qiu et al. 2015). In addition, ETH can induce PG expression in plant fruits (Wang et al. 2000). In this study, *hcc1* and *KO* lines displayed decreased ETH content and exogenous ETH supplementation can induce the expression of *HCC1* and increase PG activity (Fig. 6A–D). In contrast, 1-MCP (ETH signal blocker) repressed ETH synthesis and *HCC1* expression (Additional file 1: Fig. S9). These results demonstrated that chlorophyll metabolism was regulated by ETH in *hcc1*.

Ethylene plays a critical regulatory role in plant senescence and promotes the degradation of pectin in cell walls. During this process, PGs cleaves α-1,4-polygalacturonic acid to produce OG, including D-GA (Biely et al. 1996). OGs function as signaling molecules in plant growth and development and can induce *PG* gene expression via feedback regulation (Moscatiello et al. 2006; Savatin et al. 2011). In the current study *hcc1* revealed decreased PG activity and D-GA content and exogenous D-GA upregulates the expression of *HCC1* and enhanced ETH content (Figs. 5A, C and 7A–C). This data suggested that there may be feedback regulatory mechanism between products of pectin and ETH synthesis. As the exogenous application can lessen the deep-green leaf phenotype of *hcc1* (Fig. 7D). Overall, these results suggested that *HCC1* is involved in the chl degradation by regulating the expression of ethylene controlling genes. *Hcc1* also showed a phenotype of withered leaf tip, similar to that of previously reported *psl1*, suggesting *hcc1* is an allelic mutant to *psl1*. However, the WT of the *psl1* mutant has a darker leaf color compared to the WT of *hcc1*. This difference can be attributed to variations in *japonica* and *indica* cultivars.

**Fig. 8:**
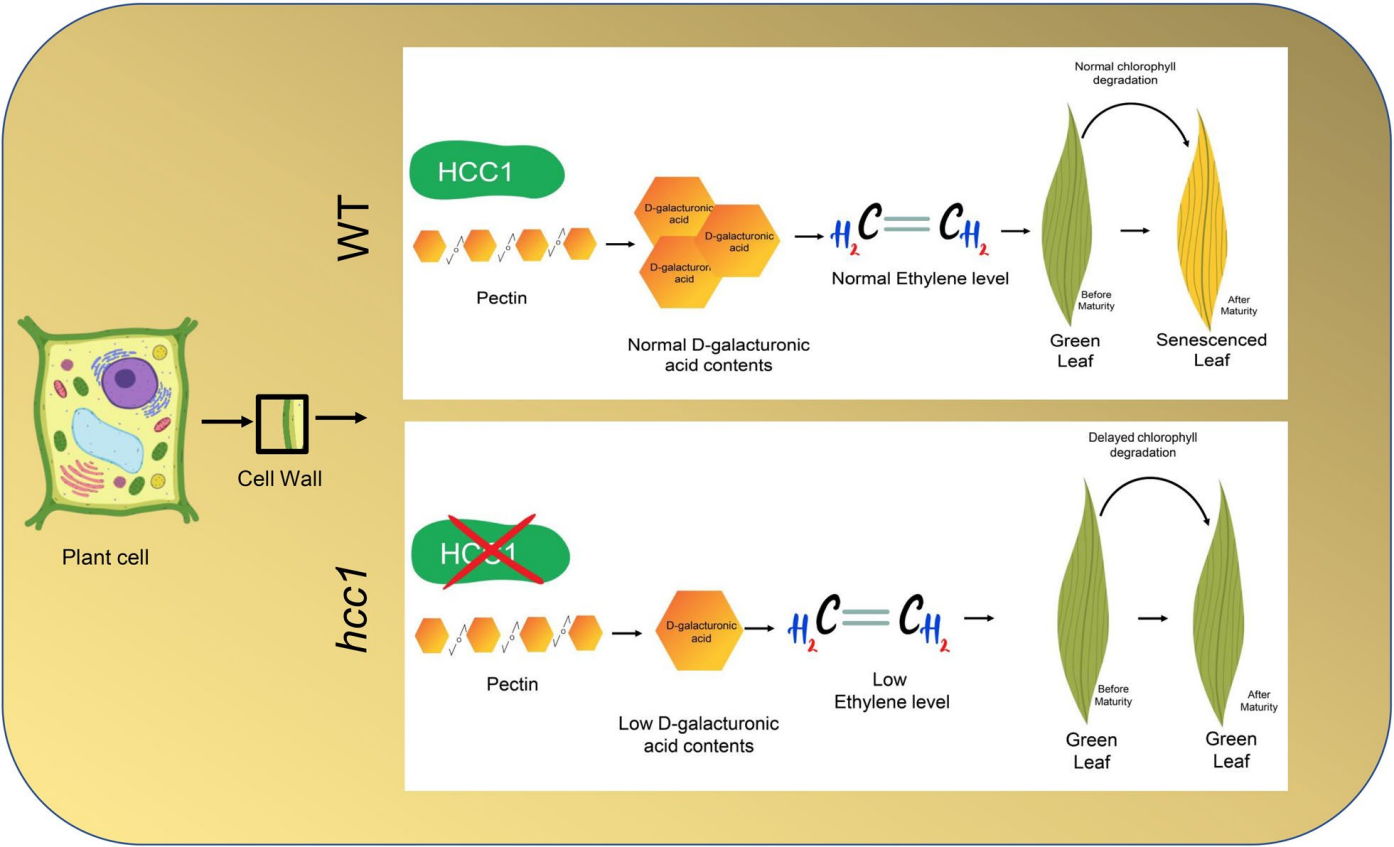
Hypothesized working model of *HCC1* in regulating leaf high chlorophyll content phenotype

Pectin is an important enzyme responsible for fruit ripening, leaf senescence and responses to various stresses. When Pectinase degrades the pectin into its products (OG) different ethylene-producing enzymes e.g., 1-aminocyclopropane-1-carboxylate (ACC) synthase and ACC oxidase are released. Previous studies revealed that these enzymes convert ACC into ethylene (Gwanpua et al. 2014). OGs also acts as signaling molecule of various pathways including ethylene biosynthesis in plants (Field 2009). Regulation through signaling pathways is usually achieved through the expression of genes involved in the ethylene. However, how exactly pectin regulates ethylene production is yet elusive. Here, we proposed a working model to show how a mutation of *HCC1* inhibited PG activity and alters the pectin metabolism due to decreased levels of D-GA content in the cell. D-GA acts as a signal molecule of ETH synthesis and delays chlorophyll degradation (Fig. 8). However, how the OGs interact with ETH and whether *HCC1* has any direct binding partner of ETH pathway are yet to be answered in the future.

## Conclusion

In the current study, we characterized a high chlorophyll content mutant *(hcc1)* that showed repression of chlorophyll degradation metabolism coupled with a significant decrease in growth and yield. Previous studies have highlighted the role of PG in cell wall modification, drought tolerance and leaf rolling, however, its effect on chlorophyll metabolism was not known so far. Map-based cloning and genetic analysis of *HCC1*, encoding a PG, validate its function in repressing the metabolism of chlorophyll degradation. The knockout lines of *HCC1* displayed phenotypes of high chlorophyll content and reduced plant growth and development, which were similar to that of *hcc1*. The phenotype of complementation was comparable with that of WT confirming it’s the function of *HCC1*. We further found that *HCC1* alters the cell wall composition and represses the PG activity. Moreover, *hcc1* also showed decreased contents of pectin and D-GA and ethylene. D-GA treatment can increase ETH contents and induces the expression of *HCC1*. Exogenous application of ETH and D-GA can successfully induce the degradation of chl in *hcc1*. Together, our data demonstrated a novel function of *HCC1* in chlorophyll degradation via the ETH pathway.

### Supplementary Information

**Additional file 1**. Fig S1 to S4.

## Data Availability

The data associated with the manuscripts are included in the supporting information file.
