# Supplementary material for: HCC1, a Polygalacturonase, Regulates Chlorophyll Degradation via the Ethylene Synthesis Pathway
Source: Rice (N Y). 2023 Dec 9;16:57. doi: 10.1186/s12284-023-00675-8 (PMC10710397; doi:10.1186/s12284-023-00675-8)
Supplement: Supplementary file 1 — Additional file 1. Fig S1 to S4. [file 12284_2023_675_MOESM1_ESM.docx]

**Supporting Information**

**Article title:** *HCC1*, a polygalacturonase, regulates pectin metabolism and chlorophyll degradation *via* the ethylene synthesis pathway

**The following supporting information is available for this article:**

**Fig. S1** Comparison of SPAD value from seedling to maturity between WT and *hcc1*.

**Fig. S2** The Manhattan plots of MutMap re-sequencing analysis.

**Fig. S3** The molecular identification of knockout lines and complementary lines.

**Fig. S4** Comparison of SPAD value from seedling to maturity between KTK and *KO1.*

**Fig. S5** Phylogenetic tree of HCC1 from different species.

**Fig. S6** Sequence alignment and domain analysis of HCC1.

**Fig. S7** The chromatogram of D-galacturomic acid (D-GA) of high-performance

liquid chromatography (HPLC).

**Fig. S8** The relative expression levels of ethylene synthesis-related genes*1-aminocyclopropeane-1*

*-carboxylic acid synthetase* (*ACSs*) and *1-aminocyclopropane-1-carboxylic acid oxidase* (*ACOs*) in leaves of WT, *hcc1* and KTK, *KO1* at maturity.

**Fig.S9** Comparison of ethylene content and *HCC1* expression in wild type

after 1-methylcyclopropene (1-MCP) treatment.

**Table S1** Primers of chlorophyll metabolism-related genes performed for quantitative real-time PCR.

**Table S2** Primers of ethylene synthesis-related genes performed for quantitative real-time PCR.

**Table S3** The markers of candidate gene mapping for PCR.

**Table S4** The list of candidate genes of *hcc1* mutant by MutMap analysis.

**Table S5** Primers of mutated-site sequence performed for PCR.

**Figure S1.** **Comparison of SPAD value from seedling to maturity between WT and *hcc1***. Ss, seedling stage; Ts, tillering stage; Bs, booting stage; Hs, headling stage; Fs, filling stage; Ms, mature stage. Mean and SD were obtained from three independent measurements. Statistical analysis was performed using Student’s *t*-test, * and** indicate *p* < 0.05 and *p* < 0.01, respectively.


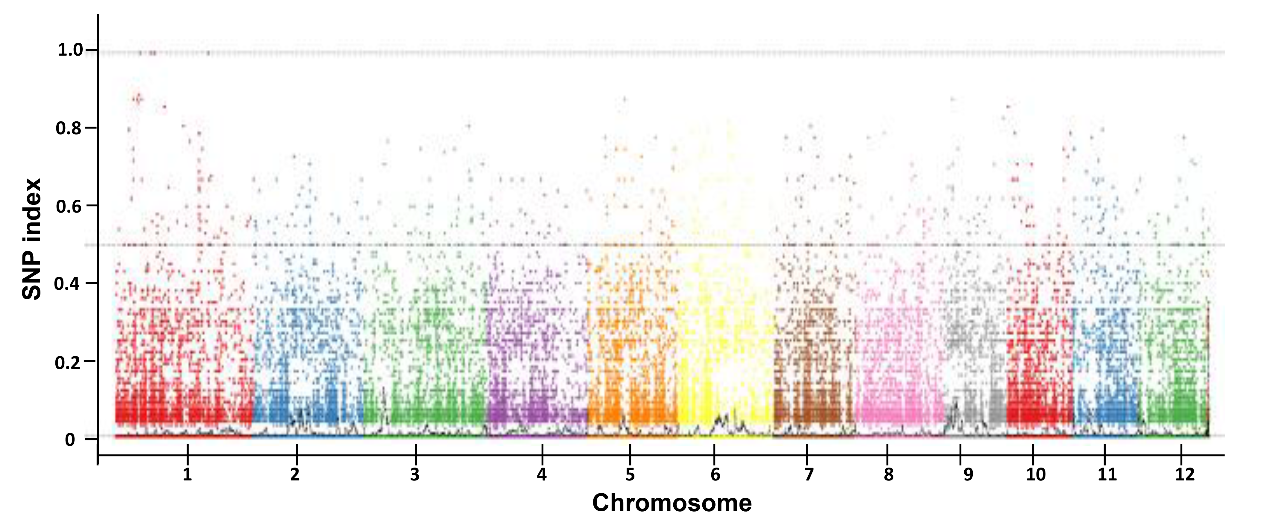


**Figure S2. The Manhattan plots of MutMap re-sequencing analysis**. The image indicates that there are four SNP, on chromosome 1, with an SNP index is 1.


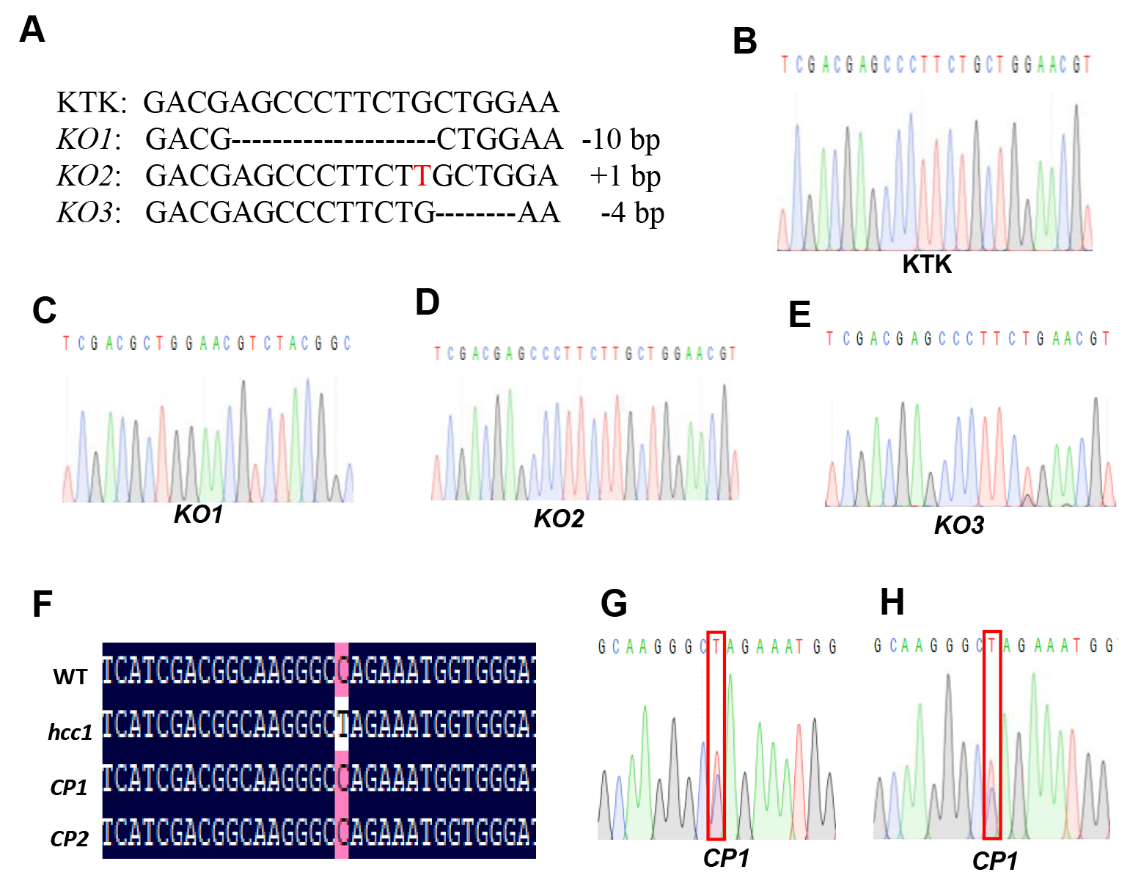


**Figure S3.** **The molecular identification of knockout lines and complementary lines.** (A) Mutation sites created through CRISPR/CAS9 *knockout line 1* (*KO1*), *knockout line 2* (*KO2*) and *knockout line 3* (*KO3*). (B-E) Chromatograms showing the sequence comparison of wild type, a japonica variety Kitaake (KTK), and knockout lines. (F) Sequence alignment of WT, *hcc1* and T_1_ *complementary line 1* (*CP1*) and *complementary line 2* (*CP2*). (G, H) Chromatograms showing the sequence of *CP1* and *CP2*.

**Figure S4. Comparison of SPAD value from seedling to maturity between KTK and *KO1***. Ss, seedling stage; Ts, tillering stage; Bs, booting stage; Hs, headling stage; Fs, filling stage; Ms, mature stage. Mean and SD were obtained from three independent measurements. Statistical analysis was performed using Student’s *t*-test, * and** indicate *p* < 0.05 and *p* < 0.01, respectively.


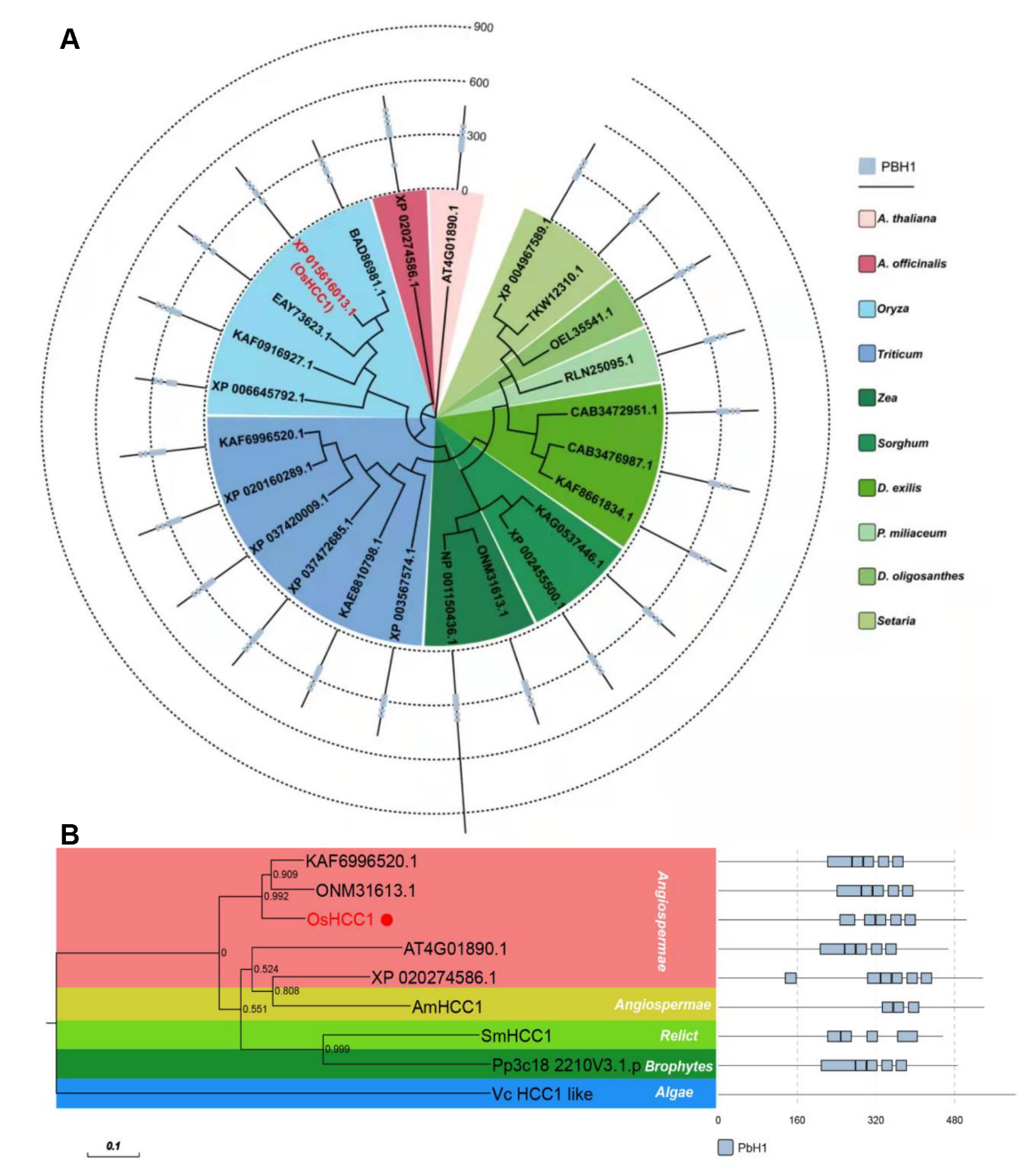


**Figure S5. Phylogenetic tree of HCC1 from different species.** This tree was generated using the MEGA 8.0.26 program by the Minimum-evolution method. PBH1, parallel beta-helix repeats. The accession number of representative protein sequence: *Triticum aestivum* L. (KAF6996520.1), *Zea mayz* L. (ONM31613.1), *Arabidopsis* (AT4G01890.1), *Asparagus officinalis* L. (XP_020274586.1), *Physcomitrium patens* (Pp3c182210v3.1.p), *Selaginella moellendorffii Hieron*. (SmHCC1), *Amborella trichopoda* (AmHCC1), *Volvox carteri* (VcHCC1 like).


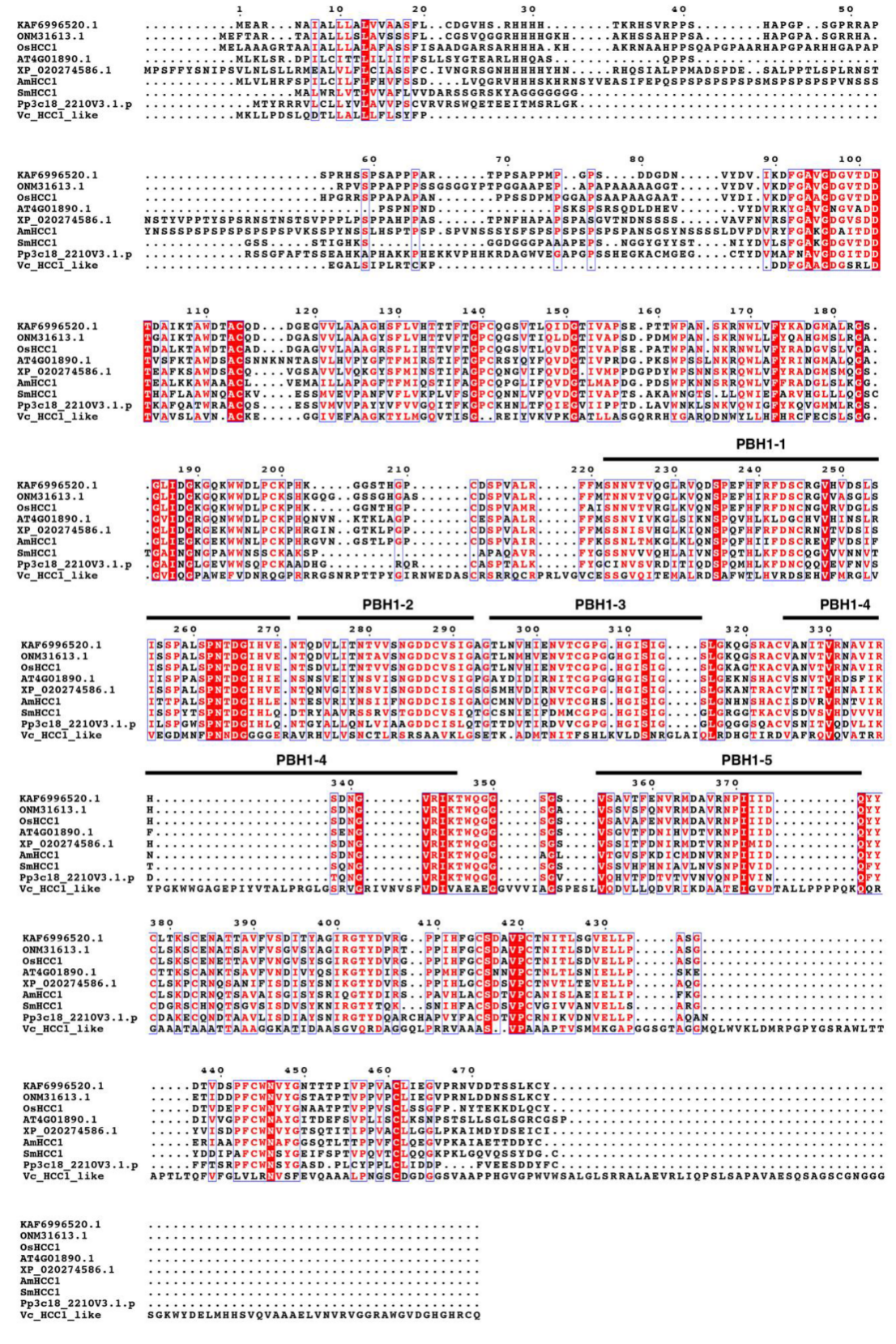


**Figure S6. Sequence alignment and domain analysis of HCC1.** The multiple sequence alignment was built by ClustalX2.1 software. PBH1, parallel beta-helix repeats. The conserved domains were identified by the online tool Smart (<http://smart.embl.de/>). The accession number of representative protein sequence: *Triticum aestivum* L. (KAF6996520.1), *Zea* *mayz* L*.* (ONM31613.1), *Arabidopsis* (AT4G01890.1), *Asparagus officinalis* L*.* (XP_020274586.1), *Physcomitrium patens* (Pp3c182210v3.1.p), *Selaginella moellendorffii* Hieron. (SmHCC1), *Amborella trichopoda* (AmHCC1), *Volvox carteri* (VcHCC1 like).


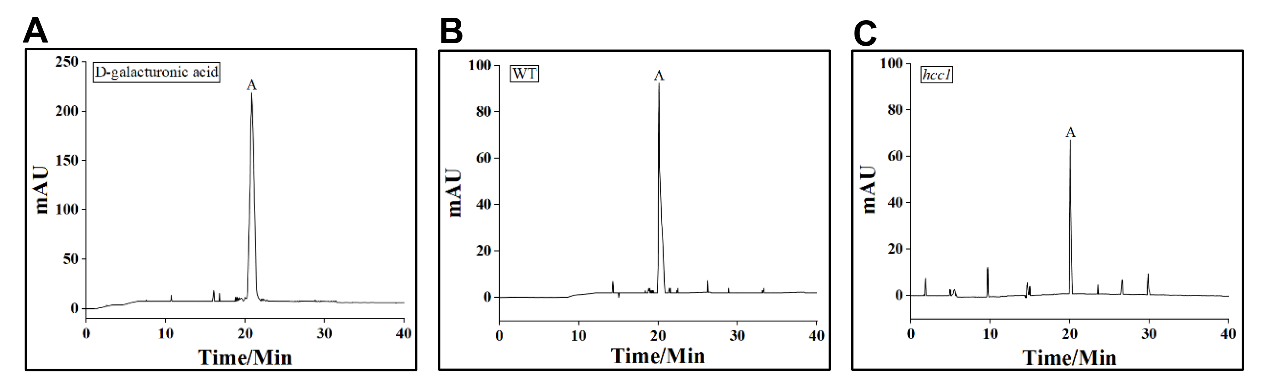


**Figure S7. The chromatogram of D-galacturomic acid (D-GA) of high-performance liquid chromatography (HPLC).** (A) The chromatogram of the standard used for D-GA analysis. (B) The chromatogram of D-GA in WT and (C) *hcc1*.


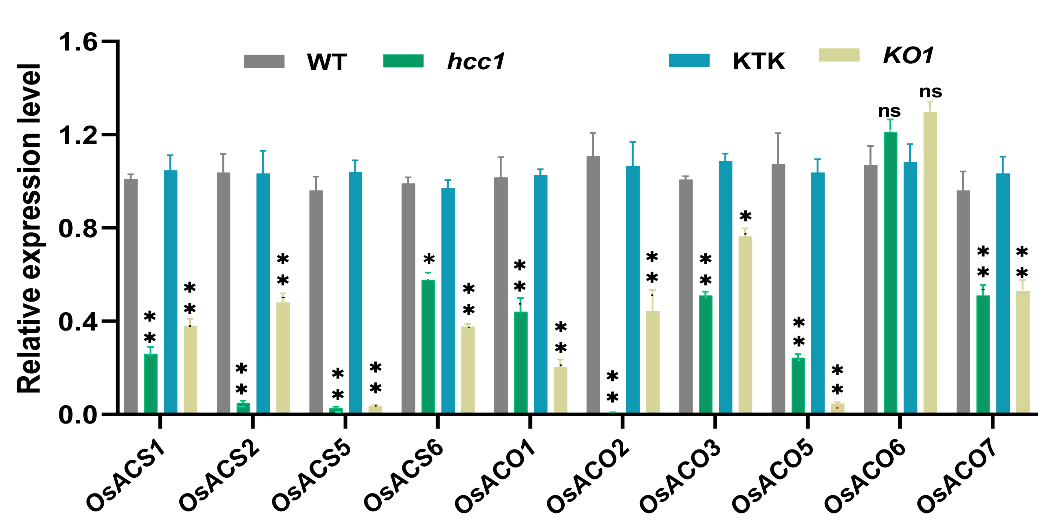


**Figure S8.** **The relative expression levels of ethylene synthesis-related genes *1-aminocyclopropeane-1-carboxylic acid synthetase* (*ACSs*) and *1-aminocyclopropane-1-carboxylic acid oxidase* (*ACOs*) in leaves of WT, *hcc1* and KTK, *KO1* at mature stage.** Mean and SD were obtained from three independent measurements. Statistical analysis was performed using Student’s *t*-test, * and ** indicate *p* < 0.05 and *p* < 0.01, “ns” indicate no significant difference.


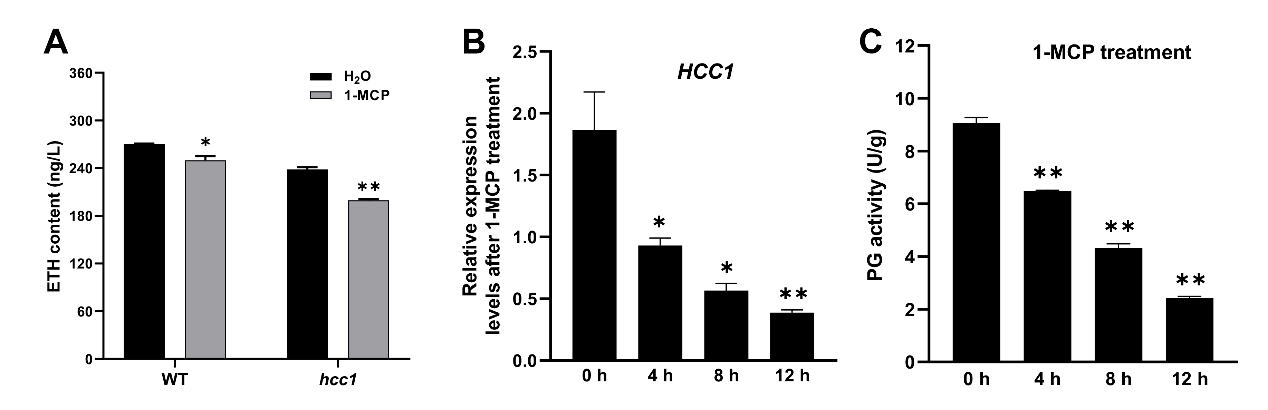


**Figure S9.** **Comparison of ethylene content and *HCC1* expression in wild type after 1-methylcyclopropene (1-MCP) treatment.** (A) Changes of ethylene (ETH) content in wild type (WT) after 1-MCP treatment. Figure B and C indicated the changes of *HCC1* expression ang its enzyme activity after 1-MCP treatment respectively. Mean and SD were obtained from three independent measurements. Statistical analysis was performed using Student’s *t*-test, * and ** indicate *p* < 0.05 and *p* < 0.01.

**Table S1. Primers of chlorophyll metabolism-related genes were performed for quantitative real-time PCR.**

| Primer | Forward primer | Reverse primer |
| --- | --- | --- |
| OsGluRS | TGACCTTATTCGTGGTGAGGTCAG | TACACTGGCTGGCCATTGCTTC |
| OsChlD | GGCTCGATTGCAAATGCTGACC | GTTAGCCAAACCCTCCTCCCATTC |
| OsChlH | AGTGGCTGAACAGCACATTGGG | CAACGCATGTGACTTCCCTGTTC |
| OsV5B | AGGGCGTCTATGACAGGGTTTG | TCACTGGGACAAGCAAAGATCCC |
| OsYGL1 | TGGCCAGGCATTATTTGGAACCC | AAGCAACCGGGAGTGACTGAAG |
| OsCAO1 | TCAGGTGTTGCTCCAGAAAGGG | GCACCGCATACACCTTGATTCCTC |
| OsHAP3H | GCCAAGATCTCCAAGGAGTCGAAG | CGCCTGTAACGAAGCTGATGAAC |
| OsNYC1 | CGCAATGTTGTCATAACTGGAAGC | AGAAGGAACTCCCGAGCAAGTG |
| OsSGR | ACGCATGCAATGTCGCCAAATG | GAGCTGAGCTAAATGCCACTACG |
| OsNOL | CAAGCAAGGAAGCACTTGATCTGG | GATTTCAGCCATAGCGGCATCG |
| OsNYC3 | TGCTGACCACTCAACGAATGTGG | TGCCTTTGGCACCTAGATAGAGC |
| OsSRLK | CCAACATGCTTCACGGAAAGAGAG | CCCTGGTTTCCCAGTTCAAAGGTG |
| OsTSD2 | GGAGAACCATTCGCGACTTTGC | AAGCACAGGGCCTGATTTCCTTG |
| OsPME1 | CGACGGCTCCACAACTTTCAAC | TGTTCTCCACCTTGAGGTCCTG |
| OsGATA12 | AAAGGAGGCAGCCATGGGAAAG | TCTCTCCCGGTTGAGATCATGC |
| OsPAO | GCTACCAAGCCTCCGATGTTAC | ACAGATCCCTCTGGATGGTCAC |
| \| OsACTIN \| \| --- \| | ACCATTGGTGCTGAGCGTTT | CGCAGCTTCCATTCCTATGAA |

**Table S2. Primers of ethylene synthesis-related genes performed for quantitative real-time PCR.**

| Primer | Forward primer | Reverse primer |
| --- | --- | --- |
| OsACTIN | ACCATTGGTGCTGAGCGTTT | CGCAGCTTCCATTCCTATGAA |
| OsACS1 | TAGTAGCAGCAGTAGCAGATTC | GGCACTAGCTTTTATTGACGAG |
| OsACS2 | GCCTTACTACGTCGACTACATT | TAGCCTAGTTTCCACAAACGAT |
| OsACS5 | CTCATCCCTACCCCTTACTACC | GAGATGAGGTGGATGTCGTTG |
| OsACS6 | ATAATGAAAGCATCGTGACAGC | TCTTGCGCAATCTCTCTCTATT |
| OsACO1 | GTTTCGTCGTCGTTTTGTGATA | GCTGCATGCTCTTTTATTCAGT |
| OsACO2 | GGTGAAAGAGAGGAACTTGAGA | GTAGTGGTCCTTGGTCATCTTC |
| OsACO3 | GGAGGAGAAGTTCAAGGAGTTC | CAAATTGCTTCATCACCTGCC |
| OsACO5 | TACATGGATGTGTACGCCAAG | AACTTTAAGCAGATTTTGGCGC |
| OsACO6 | GATGTTGCTGTTTTAGGGTCAG | CCTTCACACTGTGGTCAATTTT |
| OsACO7 | TTCCAGTGATTGATCTCAAGGG | GAGGCGTAGAATTTCTTCTCCA |

**Table S3. The markers of candidate gene mapping for PCR.**

| Primer | Forward primer | Reverse primer |
| --- | --- | --- |
| RM8068 | GTGTCATATGCAAGCAACAACTCC | AGTATGTACGTCTCCTCCGTTGC |
| RM8004 | TCTCTACCACCGTCTTCTCTCTCC | CTGAACGCAGCCACATCTAAGC |
| RM1196 | CAAGGATAAGGACATCTCTTGC | CTCAGGACCCTGCTTAGATACC |
| Indel.1 | AAAAGAGTCATCATGCAAGG | ATTGTCATATCCCATCCAAC |
| Indel.2 | GGCTCTAAGAAGGTCCTGTT | GCTGCTTGGAGGTACTGTAA |
| Indel.3 | CCCTCCGTTTGATAATGTAA | TTACACGTACTTCCTCCGTT |
| Indel.4 | CTAGAATTCAGCTCCTTCCA | TGTGTTCACTCGTCAGGTC |
| Indel.5 | AGTGAGAAACAGTGCATGAG | GGGGACATGTCTGTAACCTA |
| Indel.6 | AATGTGTGTATGACAGGTGG | ACATACACGCGTACTAGCAA |
| Indel.7 | TAGGCAACTTTCTCTCCAAG | CAAATAAGGGAATATAGCGG |
| Indel.8 | AGGCTAGATCATGACACTACAA | GCAAAAGGCACTTATGAGAC |
| Indel.9 | TACATGAATTTGCGTGTGAT | AATTGTGATTGAGGGATACG |

**Table S4.** **The list of candidate genes of *hcc1* mutant by MutMap analysis**

| Candidate genes | Variant type | Chromosome | Position | Reference | Alternative |
| --- | --- | --- | --- | --- | --- |
| LOC_Os01g12280 | nonsynonymous | 1 | 7189088 | C | T |
| LOC_Os01g19170 | Stop gain | 1 | 11827747 | C | T |
| LOC_Os01g10400 | upstream | 1 | 5876568 | C | T |
| LOC_Os01g13550 | upstream | 1 | 8209972 | C | T |

**Table S5. Primers of mutated-site sequence performed for PCR.**

| Primer | Forward primer | Reverse primer |
| --- | --- | --- |
| HCC1-ms | ATCCACACCACCGTCTTCAC | ACTCACCACTGGGCTGTCAC |
